# Comorbidities and factors associated with central nervous system infections and death in non-perinatal listeriosis: a clinical case series

**DOI:** 10.1186/s12879-016-1602-3

**Published:** 2016-06-07

**Authors:** C. Maertens De Noordhout, B. Devleesschauwer, A. Maertens De Noordhout, J. Blocher, J. A. Haagsma, A. H. Havelaar, N. Speybroeck

**Affiliations:** Institute of Health and Society (IRSS), Université catholique de Louvain, Clos Chapelle-aux-Champs, 30 bte B1.30.15, Brussels, 1200 Belgium; Ghent University, Merelbeke, Belgium; University of Florida, Gainesville, Florida USA; University Department of Neurology, CHR Citadelle, Liège, Belgium; Department of Neurology, University Medical Center, Göttingen, Germany; Department of Public Health, Erasmus MC, Rotterdam, The Netherlands; Utrecht University, Utrecht, The Netherlands

**Keywords:** Listeriosis, Comorbidities, Central nervous system infections, Death, Clinical series

## Abstract

**Background:**

Listeriosis is a rare disease caused by the bacterium *Listeria monocytogenes* and mainly affects at risk people. Listeriosis can lead to sepsis, central nervous system (CNS) infections and death. The objectives of this study were to describe and quantify comorbidities and neurological sequelae underlying non-perinatal listeriosis cases and to describe the factors associated with death and CNS infections in non-perinatal listeriosis.

**Methods:**

We retrospectively collected clinical data through computerized, paper or microfilmed medical records in two Belgian university hospitals. Logistic regression models and likelihood ratio tests allowed identifying factors associated with death and CNS infections.

**Results:**

Sixty-four cases of non-perinatal listeriosis were included in the clinical case series and 84 % were affected by at least one comorbid condition. The main comorbidities were cancer, renal and severe cardio-vascular diseases. Twenty-nine patients (45 %) suffered from a CNS infection and 14 patients (22 %) died during hospitalization, among whom six (43 %) had a CNS involvement. Among surviving patients, eleven suffered from neurological sequelae (22 %) at hospital discharge; all had CNS infection. Five of these patients (45 %) still suffered of their neurological sequelae after a median follow-up of one year (range: 0.08–19). The factor associated with death during the hospitalization was the presence of a severe cardiovascular disease (OR = 4.72, *p* = 0.015). Two factors inversely related with CNS infections were antibiotic monotherapy (OR = 0.28, *p* = 0.04) and the presence of renal disease (OR = 0.18, *p* = 0.02).

**Conclusions:**

In a public health context these results could be a starting point for future burden of listeriosis studies taking into account comorbidity.

**Electronic supplementary material:**

The online version of this article (doi:10.1186/s12879-016-1602-3) contains supplementary material, which is available to authorized users.

## Background

Listeriosis is caused by *Listeria monocytogenes*, a gram-positive bacterium that was first described by Murray *et al*. in rabbits and guinea pigs in 1926 [1]. The bacterium is typically widespread in nature but the main sources of infection are mainly dairy and ready-to-eat foods [2]. *L. monocytogenes* is a high concern for the food industry because it has the capacity to survive and grow in harsh conditions such as wide pH ranges, high salt concentrations and at refrigeration temperatures [3].

Listeriosis is a rare disease showing a high case-fatality ratio. It was estimated that in 2010, listeriosis caused 23,150 illnesses (95 % Credible Interval [CrI]: 6,061-91,247) and 5,463 deaths (95 % CrI: 1,401-21,497) worldwide, resulting in 172,823 Disability-Adjusted Life Years (DALYs) (95 % CrI: 42,079-679,465) [4].

Listeriosis mainly affects at risk groups such as pregnant women, the elderly, and patients affected by comorbidities such as cancer or transplantation [5]. Comorbidity can be defined as the presence of any clinical condition which qualifies for formal classification as a disease additional to listeriosis. Three types of comorbidities can be distinguished: 1) unrelated (diseases/conditions happening by chance on the same patient); 2) indirectly related (diseases/conditions with common risk factors while pathophysiology is unrelated); and 3) directly related (when pathophysiology shows that one condition can be regarded as natural consequence or parallel manifestation of the other condition) [6].

Listeriosis typically affects patients with underlying diseases and can cause severe symptoms such as sepsis or central nervous system (CNS) infections, possibly leading to lifelong consequences or death [5, 7]. It is therefore important to identify high risk populations and identify risk factors linked with CNS infections and death caused by listeriosis.

The aims of our study were 1) to identify and quantify the comorbidities underlying listeriosis cases, 2) to describe the factors associated to death and CNS infections and 3) and to describe and quantify the neurological sequelae associated to non-perinatal listeriosis.

## Methods

### Data collection

We collected information about listeriosis cases in two Belgian university hospitals: ‘Le centre hospitalier universitaire’ (CHU) in Liège in the period 2003-2013 and ‘Les cliniques universitaires saint Luc’ (UCL) in Brussels in the period 1978-2014 (April). We collected retrospective data through computerized, paper or microfilmed medical records. We extracted general information (date of hospital and discharge admission), information on the patients characteristics (age, gender, weight, height), information on medical and surgical antecedents, characteristics of the patient at admission, blood and cerebrospinal fluid culture characteristics at admission, treatment received against listeriosis, outcome of listeriosis and information about follow-up.

The listeriosis cases were detected by laboratories included in each hospital and listeriosis was defined when Listeria monocytogenes was isolated from a sterile site (blood, cerebrospinal fluid or aortic wall).

All collected data are available in Additional file 1.

### Statistical analyses

We evaluated the association between death during hospitalization and CNS infections, and various potential risk factors based on literature review, i.e., age at hospital admission, presence of a severe cardiovascular disease, antibiotic monotherapy, gender, blood pressure, diabetes mellitus, immunosuppressive therapy, cancer, alcoholism, renal disease, HIV infection, hepatic disease, auto-immune disease, immunocompromised status, neutrophil and lymphocyte count in blood, C-Reactive Protein (CRP), creatinine, and blood platelets, using univariable logistic regression analyses. All covariates with a p-value <0.10 were subsequently included in a multivariable logistic regression model. The two final models were chosen based on the Likelihood Ratio Test using a backward inclusion methodology, testing for the significance of elimination of the variable at each stage. We only retained significant variables in the final models. For death we started with a full model including antibiotic monotherapy, age at admission, hepatic disease, severe cardiovascular disease, renal disease and transplantation. For CNS infection we started with a full model including antibiotic monotherapy, immunosuppressive therapy and renal disease.

We performed a t-test for equal variances to compare mean age in antibiotic bi-therapy and antibiotic monotherapy groups.

We applied a Mann-Whitney test to compare time until antibiotic therapy between CNS infection cases and non CNS infection cases.

We performed the analyses with SPSS 22.0 [8].

## Results

**Fig. 1:**
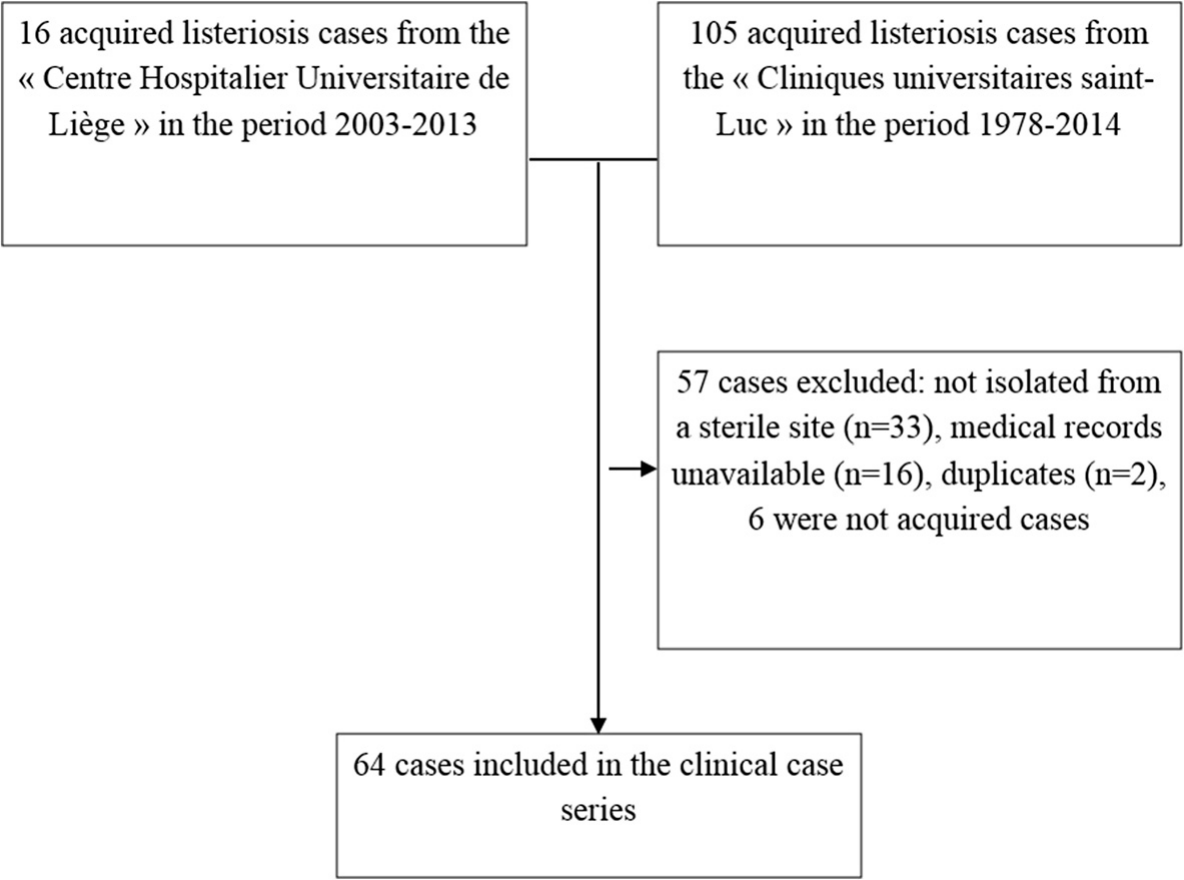
Selection of cases

We identified 16 non-perinatal listeriosis cases in CHU and 105 listeriosis cases in UCL. Fifty-seven cases were excluded from the analysis (33 were not isolated from a sterile site, 16 medical records were unavailable because too old, 2 were duplicates, 6 were perinatal cases) (Fig. 1).

**Fig. 2:**
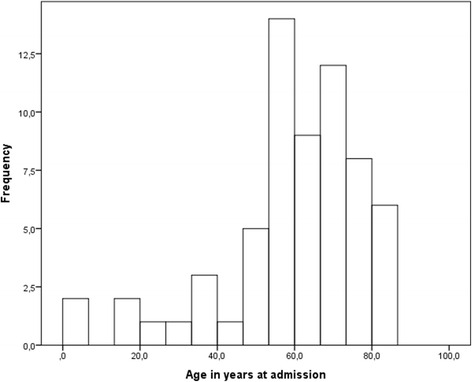
Age distribution of patients (*n* = 64)

Among the remaining 64 cases, 34 patients were male (53 %) and the median age was 62 years (Interquartile interval 53-72) (Fig. 2) (Additional file 1).

**Fig. 3:**
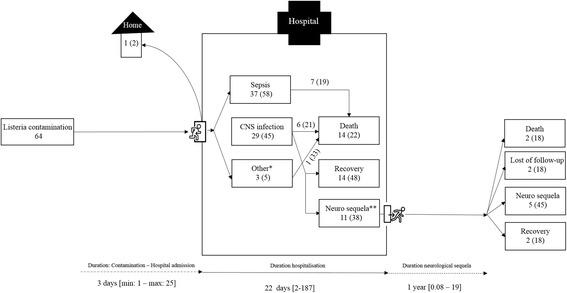
Patients follow-up after positive listeria culture. * Peritonitis – endocarditis - infrarenal abdominal aortic aneurysm. ** 1) bilateral hearing loss, 2) discrete dysarthria and mild instability in Romberg (test used to investigate the ataxia. A positive Romberg test suggests that the ataxia is sensory in nature) and in walking on a straight line, 3) slight diplopia (a trochlear nerve palsy), 4) memory loss and forgetfulness (damage of the mammillary body and fornix), 5) dystonia (difficulty in opening jaw), 6) persistence of an impaired general condition with small steps walk and brachypsychie, 7) discrete right dysdiadochokinesia, hypoesthesia in the territory V2, V3 to and an ataxic walk with deviation to the right side, 8) right hemibody motor deficit with Babinski sign, 9) dysarthria, 10) deep sensitivity deficit of the left arm and left leg and 11) mild paresis of the right upper and lower limb

All patients, except one, were hospitalized. The median duration of hospitalization was 22 days (range: 2–187) (Fig. 3). The exact source of contamination was never identified, only one patient reported having eaten camembert but the contamination of the cheese was not analyzed. Thirteen patients (20 %) were previously hospitalized within the four weeks of hospital admission, which could suggest a nosocomial infection.

### Clinical data

We found that among the 64 people affected by acquired listeriosis, most frequent comorbidities were ongoing immunosuppressive treatment (53 %), cancer (34 %), severe cardiovascular disease (30 %) and renal disease (27 %). 84 % of the patients were affected by at least one comorbidity (Table 1). One patient was 3 months pregnant at admission.

**Table 1:** Comorbidities and factors associated with listeriosis cases (*n* = 64)

Comorbidity	N	%
Immunosuppressive therapy	34	53.1
Cancer	22	34.4
Severe cardiovascular disease	19	29.7
Renal disease	17	26.6
Transplantation^a^	12	18.8
Hepatic disease	11	17.2
No recognized comorbidities	10	15.6
Diabetes mellitus	8	12.5
HIV	6	9.4
Auto-immune disease	5	7.8
Alcoholism	3	4.7
Pregnancy	1	1.6

^a^ Kidney (6), heart (5), Liver (1)

The immunosuppressive treatments were anti-rejection treatment (*n* = 13, 32 %), chemotherapy (*n* = 11, 27 %), radiotherapy (*n* = 1, 2 %), corticosteroids (*n* = 13, 32 %), infliximab (*n* = 2, 5 %). The encountered cancers were colon (*n* = 3, 14 %), liver (*n* = 3, 14 %), breast (*n* = 3, 14 %), lung (*n* = 2, 10 %), leukemia (*n* = 5, 24 %), myeloma (*n* = 1, 5 %), lymphoma (*n* = 3, 14 %) and melanoma (*n* = 1, 5 %). The severe cardiovascular diseases (*n* = 19) were heart transplantation, heart transplantation followed by a triple bypass of the transplanted heart followed by high blood pressure, heart transplantation followed by multiple myocardial infractions and high blood pressure, double heart transplanted, heart transplantation, angina pectoris and high blood pressure treated with cardiac bypass, angina pectoris, ischemic heart disease followed by a heart transplantation and followed by an aortic bypass, myocardial infarction caused by a ischemic heart disease, myocardial infarction followed by cardiac decompensation, obstructive hypertrophic heart disease and essential hypertension treated by pacemaker, obstructive hypertrophic cardiomyopathy, cardiac decompensation, cardiac decompensation treated by pacemaker, cardiac decompensation treated by bypass but remaining high blood pressure, aortic insufficiency, aortic dissection and high blood pressure, aortic insufficiency, aortic insufficiency.

The renal diseases were kidney transplantation (*n* = 6, 35 %), chronic renal disease (*n* = 7, 41 %), polycystic kidney (*n* = 1, 6 %), end stage renal (dialysis) disease (*n* = 2, 12 %), Raynaud disease with cystic kidney (*n* = 1, 6 %). The hepatic diseases were congestive hepatopathy (*n* = 2, 20 %), chronic hepatitis (*n* = 1, 10 %), hepatitis C (*n* = 4, 40 %), liver transplantation (*n* = 1, 10 %), liver cancer (*n* = 2, 20 %).

Sixty-four percent of the patients were immunocompromised by treatment, cancer or HIV.

Nine patients (14 %) did not suffer from any comorbidity. Among these patients the median age was 41.5 (range: 1.3-75.1), 7 patients (78 %) had CNS infection, 6 (67 %) had neurological sequelae at hospital discharge. One died 19 years after hospital discharge.

Among all cases, the most frequent symptoms at admission were fever (>38 °C) (77 %), diarrhea (23 %) and vomiting (13 %). For 20 patients, the median duration between appearance of symptoms and admission at hospital was 3 days (range: 1–25). Two patients were infected during their hospitalization after 26 and 11 days respectively (Fig. 3). The first patient was a man hospitalized for a heart transplantation and the second was a woman hospitalized for a breast implant removal.

*L. monocytogenes* caused a central nervous system infection in 29 patients (45 %). Twenty-seven neurological cases were confirmed by positive CSF culture and two by neuroimaging. The mean CSF protein level was 252.6 (sd 227.8) mg/L, the median CSF lymphocytes percentage was 40 (range: 2-88) and the median CSF monocytes percentage was 6 (range: 5.5-6).

Among the neurological cases, the classical triad for meningitis of neck stiffness, fever and altered mental status were present among eight (28 %) of cases. In addition, 14 (48 %) had headaches, five (17 %) seizures, six (21 %) were aphasic, six (21 %) showed hemiparesis, seven (24 %) had cranial nerve palsies, four (14 %) had nystagmus, five (17 %) suffered from dysarthria and seven (24 %) had limb and gait ataxia.

The initial reported listeriosis manifestations were: sepsis for 37 (58 %) patients, endocarditis for one (2 %), peritonitis for one (2 %), infrarenal abdominal aortic aneurysm for one (2 %), meningitis for 14 (22 %), meningo-encephalitis for ten (19 %) and rhombo-encephalitis for one (2 %). One patient suffered from both brain abscess and rhombo-encephalitis (2 %), one patient suffered from both brain abscess and meningo-encephalitis (2 %), one patient suffered from both brain abscess and encephalitis (2 %) and one patient suffered from both brain abscess and meningitis (2 %).

Five patients (8 %) suffered from both meningitis and sepsis, four patients of meningo-encephalitis and sepsis (6 %), one of encephalitis and sepsis (2 %). For one patient who suffered of sepsis, *L. monocytogenes* was also isolated from his hip prosthesis joint (Fig. 3).

In total 56 patients (92 %) received a parenteral antibiotic therapy during hospitalization. Among the 56 patients, 59 % received a bi-therapy for against listeriosis, mostly ampicillin and with gentamycin. We did not find any significant association between age at admission and the administration of parenteral antibiotic bi-therapy (*p* = 0.738). Four neurological cases (14 %) received a neurosurgical therapy: three external ventricular derivations of the cerebral fluid and one ventriculostomy. No patient who developed brain abscesses was operated.

If we assume that antibiotic therapy was implemented at admission, we did not find significant difference of time until antibiotic therapy between CNS infection cases (median 3.5 days, min-max 1-8 days) and non CNS infection cases (median 3 days, min-max 0-25) (U Mann-Whitney 53, *p* = 0.943).

The patient with endocarditis was initially treated against *Candida* (fluconazole). Then after he underwent an explanation of the left aorto-femoral prosthesis and of the left crossed femoral prosthesis and an implementation of a homograft left axillary-femoral. The patient died during hospitalization.

The patient with a *L. monocytogenes* aneurysm was successfully operated for a replacement of the abdominal aorta (cadaveric allograft) during the hospitalization.

The patient for whom *L. monocytogenes* was isolated from the hip prosthesis joint was successfully treated with parenteral gentamycin associated with a surgical scrub of the hip prosthesis.

During hospitalization 14 patients (22 %) died, among whom 6 (38 %) had a CNS infection. Listeriosis was linked to all death at hospital.

### Factors associated with CNS infections and death

In the univariate analysis, the factors associated with neurological listeriosis were antibiotic monotherapy (OR = 0.31, 95 % CI 0.10-0.93, *p* = 0.036) and the presence of a renal disease (OR = 0.27, 95 % CI 0.08-0.95, *p* = 0.042) (Table 2). Both variables were retained by the multivariable model (presence of a renal disease (OR = 0.18, 95 % CI 0.04-0.79, *p* = 0.020), antibiotic monotherapy (OR = 0.28, 95 % CI 0.09-0.92, *p* = 0.04).

**Table 2:** Comorbidities and factors associated with neurological listeriosis - Univariate logistic analysis

	Neurological listeriosis (*n* = 29)		Not Neurological listeriosis (*n* = 35)			
	N	%	N	%	OR	95 % CI
Monotherapy (*n* = 57)	11	42.3	23	71.9	0.31	0.10-0.93**
Male	14	48.3	20	57.1	0.70	0.26-1.88
Saint Luc hospital	23	79.3	29	82.9	0.79	0.27-2.79
Diabetes mellitus	2	6.9	6	17.1	0.36	0.07-1.93
Immunosuppressive therapy	12	41.4	22	62.9	0.42	0.15-1.14
Cancer	11	37.9	11	31.4	1.33	0.47-3.76
Alcoholism	1	3.4	2	5.7	0.59	0.05-6.85
Renal disease	4	13.8	13	37.1	0.27	0.08-0.95**
HIV infection	2	6.9	4	11.4	0.57	0.10-3.38
Transplantation	3	10.3	9	25.7	0.33	0.08-1.37
Severe cardiovascular disease	6	20.7	13	37.1	0.44	0.14-1.37
Hepatic disease	3	10.3	8	22.9	0.40	0.09-1.63
Auto-immune disease	1	3.4	4	11.4	0.28	0.03-2.63
Immunocompromised status	16	55.2	25	71.4	0.49	0.18-1.38
	Mean	SD	Mean	SD	OR	95 % CI
Age in years at admission	57.6	19.5	60.6	19.1	0.99	0.97-1.02
BMI (*n* = 33)	23.6	5.6	24.8	5.3	0.96	0.83-1.10
Systole at admission (mmHg) (*n* = 55)	129.2	32.9	123.3	30.2	1.01	0.99-1.03
Diastole at admission (mmHg)	75.0	12.1	70.8	19.1	1.02	0.98-1.05
% neutrophil in blood at admission (*n* = 41)	74.1	25.3	74.0	19.1	1.00	0.97-1.03
% lymphocytes in blood at admission (*n* = 37)	15.5	17.6	14.0	9.3	1.01	0.96-1.06
CRP mg/dl at admission (*n* = 47)	31.9	33.5	34.2	18.3	0.10	0.99-1.01
Creatinine in blood at admission mg/L (*n* = 60)	2.8	3.0	4.6	9.9	0.96	0.86-1.06
Blood platelets at admission/L (10^3^) (*n* = 56)	209.8	95.4	254.8	372.0	1.00	0.99-1.00
	Median	Range	Median	Range	OR	95 % CI
% neutrophil in CSF (*n* = 20)	71.5	35.0-94.0	79.0	8.5-94.6	1.00	0.96-1.04
% lymphocytes in CSF (*n* = 8)	40.0	2.0-88.0	/	/	/	/
Glucose in CSF (mg/dL) (*n* = 10)	31.0	0.4-74.0	/	/	/	/
Protein in CSF (mg/L) (*n* = 14)	164.5	27.0-732.0	/	/	/	/
Admission year	1998	1983-2013	2006	1978-2014	0.95	0.90-1.02

**p-value < 0.05

In the univariate analysis, the factors associated with death during the hospitalization were the presence of a severe cardiovascular disease (OR = 4.73, 95 % CI 1.35-16.54, *p* = 0.015), the presence of a hepatic disease (OR = 4.07, 95 % CI 1.02-16.30, *p* = 0.047) and the presence of a renal disease (OR = 4.0, 95 % CI 1.14-14.05, *p* = 0.031) (Table 3). At a multivariable level and based on the likelihood ratio test only the presence of a severe cardio-vascular disease (OR = 4.73, 95 % CI 1.35-16.54, *p* = 0.015) was significant.

**Table 3:** Comorbidities and factors associated with death at hospital - Univariate logistic analysis

	Death (*n* = 14)		Alive (*n* = 50)			
	N	%	N	%	OR	95 % CI
CNS infection	6	42.3	23	46.0	0.88	0.27-2.91
Monotherapy (*n* = 57)	7	77.8	27	56.3	2.72	0.51-14.49
Male	9	64.3	25	50.0	1.80	0.53-6.13
Saint Luc hospital	12	86.0	40	80.0	1.50	0.29-7.81
Diabetes mellitus	2	14.3	6	12.0	1.22	0.22-6.85
Immunosuppressive therapy	8	57.1	26	52.0	1.23	0.37-1.07
Cancer	7	50.0	15	30.0	2.33	0.70-7.82
Alcoholism	0	0.0	3	6.0	/	/
Renal disease	7	50.0	10	20.0	4.00	1.14-14.48**
HIV infection	1	7.1	5	10.0	0.69	0.07-6.46
Transplantation	5	35.7	7	14.0	3.41	0.88-13.22
Severe cardiovascular disease	8	57.1	11	22.0	4.73	1.35-16.54**
Hepatic disease	5	35.7	6	12.0	4.07	1.02-16.30**
Auto-immune disease	0	0.0	5	10.0	/	/
Immunocompromised status	11	78.6	30	60.0	2.44	0.61-9.88
	Mean	SD	Mean	SD	OR	95 % CI
Age in years at admission	67.9	9.9	56.8	20.5	1.04	0.98-1.09
BMI (*n* = 33)	25.4	3.0	24.0	5.8	1.05	0.90-1.23
Systole at admission (mmHg) (*n* = 55)	116.7	20.6	127.5	32.8	0.99	0.97-1.01
Diastole at admission (mmHg)	68.7	10.5	73.3	17.5	0.98	0.94-1.03
% neutrophil in blood at admission (*n* = 41)	69.7	30.8	75.1	18.9	0.99	0.96-1.02
% lymphocytes in blood at admission (*n* = 37)	11.7	9.5	15.2	13.7	0.97	0.89-1.06
CRP mg/dl at admission (*n* = 47)	55.1	110.5	28.1	32.3	1.01	0.96-1.02
Creatinine in blood at admission mg/L (*n* = 60)	7.1	15.5	3.0	3.6	1.06	0.97-1.16
Blood platelets at admission/L (10^3^) (*n* = 56)	170.7	86.3	248.9	314.5	0.98	0.99-1.01
% neutrophil in CSF (*n* = 20)	83.8	11.5	67.4	23.8	1.06	0.94-1.19
	Median	Range	Median	Range	OR	95 % CI
Admission year	2004	1984-2013	2003	1978-2014	1.01	0.95-1.07

**p-value < 0.05

### Neurological sequelae

Among the surviving patients, eleven (22 %) suffered from at least one sequela at discharge. The median age of patients affected by neurological sequelae was 51.1 (range 83.1) years. Among these patients, six (54 %) did not suffer of any recognized comorbidity but all suffered of CNS infection. One of the two children included in the study suffered of neurological sequelae.

The neurological sequelae were: bilateral hearing loss, discrete dysarthria and mild instability in Romberg (test used to investigate the ataxia. A positive Romberg test suggests that the ataxia is sensory in nature) and in walking on a straight line, slight diplopia (a trochlear nerve palsy), memory loss and forgetfulness (damage of the mammillary body and fornix), dystonia (difficulty in opening jaw), persistence of an impaired general condition with small steps walk and brachypsychie, discrete right dysdiadochokinesia, hypoesthesia in the territory V2, V3 to and an ataxic walk with deviation to the right side, right hemibody motor deficit with Babinski sign, dysarthria, deep sensory deficit of the left arm and left leg and mild paresis of the right upper and lower limb.

Among these eleven patients, two were lost to follow-up, two died (of a ischemic heart disease and of an unknown cause) after 3 and 19 years of follow-up and five still suffered from their neurological sequelae at the last contact with neurologist (median follow-up: 1 [range: 0.08–19] years (Fig. 3).

## Discussion

Among the 64 patients, 84 % suffered from an underlying disease. The main comorbidities affecting non-perinatal listeriosis cases were cancer, renal and severe cardio-vascular diseases and undergoing immunosuppressive therapy. In total, 19 % of the patients included in the clinical case series were transplanted and one patient was infected only six days after the cardiac transplantation. In all cases, the source of infection was unknown. However 20 % of the patients were previously hospitalized for 4 weeks before admission which could suggest that the infection was nosocomial. The incubation time of *L. monocytogenes* is very variable which makes the identification of the source of infection difficult [9].

Despite most non-perinatal listeriosis cases are people with comorbid conditions, the study also indicated that neurological listeriosis can affect people without (known) underlying diseases and very young immunocompetent children. Other recent case reports also revealed that listeriosis can affect very young children and that an appropriate treatment at the onset, based on intravenous ampicillin in the first line therapy, can avoid the spread of the infection in the patient [10–13].

To our knowledge this is the first study identifying factors associated with death and CNS infection. We identified that people who received a parenteral antibiotic monotherapy suffered a CNS infection less frequently than people who received a combination therapy (ampicillin and an aminoglycoside). An explanation could be that patients with neurological listeriosis have more severe initial clinical symptoms and therefore received more often broad-spectrum antibiotic therapy at admission. Although combination therapy is often promoted in patients older than 50 years, we did not find any significant association between age at admission and the administration of parenteral antibiotic monotherapy [14]. Time until antibiotic therapy is started could also be a significant factor of CNS infection. We collected information on time of appearance of symptoms for 20 patients. If we assume that antibiotic therapy was implemented at admission, we did not find significant difference of time until antibiotic therapy between CNS infection cases and non CNS infection cases.

We also identified that patients with a renal disease suffered less frequently (OR = 0.18, *p* = 0.020) from a CNS infection. One explanation could be that people suffering from a chronic renal disease (*n* = 7) receive a prophylactic antibiotic therapy that may protect against CNS infection. We also found that severe cardiovascular (OR = 4.72, *p* = 0.015) disease was the main factor associated with death caused by listeriosis. However, given the small sample size of our study, these estimates should be interpreted with caution and in the context of any other available information [15].

Moreover an acute severe illness (e.g. cardiovascular diseases) was a strong risk factor for mortality at the hospital.

In a public health context our findings could improve listeriosis burden estimations because we present rare data on type and duration of sequelae caused by CNS infections associated with listeriosis, both crucial parameters in the calculation of the number of years lived with disability, one of the two components included in the DALY calculation [16, 17]. We also think that these results could be a starting point for future burden of listeriosis studies aiming to take comorbidities into account. Ignoring comorbidities, as is the case in many burden of disease studies, could overestimate the overall disease burden [6], especially in the context of an ageing population. Correcting for comorbidities could be relevant to support policy intervention strategies. Strategies for the estimation of the prevalence and severity of multimorbid conditions do exist [18]. However, these strategies typically assume independence between the occurrence of different diseases and symptoms. Our estimates show that this is not true for listeriosis and its comorbidities.

The main limitation of our study may be that it is not necessarily representative of all non-perinatal listeriosis cases in Belgium, as the data came from two Belgian university hospitals. Both hospitals were located in Brussels and the Walloon region, excluding Flanders. However, ‘Les Cliniques Universitaires saint Luc’, counting 979 beds, is the third biggest hospital in Belgium and ‘Le Centre Hospitalier Universitaire’, counting 595 beds, is the second biggest hospital in the Walloon region. The two hospitals are also affiliated with a university and probably received most severe listeriosis cases. Finally, we expect that patients suffering of a CNS infection will be frequently transferred to a university hospital because of the need of intensive care making the representativeness of our sample reasonable.

Another weakness of our study may be the retrospective design and the long observation duration (>40 years). However, listeriosis is a rare disease making the use of a prospective study design difficult. A retrospective design and long observation duration allowed us to collect a study population sufficiently large to perform a parametric statistical analysis.. Moreover, we did not identify a significant effect of year at hospital admission on death and on CNS infection. The broad categories of comorbidities allowing statistical analysis could also be a limitation for clinicians and public health professionals to easily focus on a specific disease or health condition. To remedy this, we described each case included in disease groups (e.g. severe cardiovascular diseases) in detail.

## Conclusion

In a public health context our study could improve burden of listeriosis estimations because we proposed rare data on type and duration of sequelae caused by CNS infection associated with listeriosis, both crucial parameters in the calculation of the number of healthy life years lost by a disability, one of the two components included in the DALY calculation. Our results could also be an initial contribution to future burden of listeriosis studies aiming to take into account multi-morbidity in summary measure of population health. Additional data on neurological sequelae and comorbidities underlying listeriosis collected through other retrospective studies could be added to the data at hand to improve our estimations.

## Additional file

Additional file 1: Data on non-perinatal listeriosis cases collected in two Belgian university hospitals: ‘Le centre hospitalier universitaire’ (CHU) in Liège in the period 2003-2013 and ‘Les cliniques universitaires saint Luc’ (UCL) in Brussels in the period 1978-2014 (April). (XLSX 35 kb)
